# Depletion of pro-oncogenic *RUNX2* enhances gemcitabine (GEM) sensitivity of *p53*-mutated pancreatic cancer Panc-1 cells through the induction of pro-apoptotic TAp63

**DOI:** 10.18632/oncotarget.12433

**Published:** 2016-10-04

**Authors:** Toshinori Ozaki, Mizuyo Nakamura, Takehiro Ogata, Meijie Sang, Hiroyuki Yoda, Kiriko Hiraoka, Meixiang Sang, Osamu Shimozato

**Affiliations:** ^1^ Laboratory of DNA Damage Signaling, Chiba Cancer Center Research Institute, Chiba, Japan; ^2^ Department of Regenerative Medicine, Graduate School of Medicine and Pharmatheutical Science, University of Toyama, Toyama, Japan; ^3^ Laboratory of Cancer Genetics, Chiba Cancer Center Research Institute, Chiba, Japan; ^4^ Research Center, Fourth Hospital of Hebei Medical University, Shijiazhuang, China

**Keywords:** gemcitabine, mutant p53, pancreatic cancer, RUNX2, TAp63

## Abstract

Recently, we have described that siRNA-mediated silencing of runt-related transcription factor 2 (RUNX2) improves anti-cancer drug gemcitabine (GEM) sensitivity of *p53*-deficient human pancreatic cancer AsPC-1 cells through the augmentation of p53 family TAp63-dependent cell death pathway. In this manuscript, we have extended our study to *p53*-mutated human pancreatic cancer Panc-1 cells. According to our present results, knockdown of mutant *p53* alone had a marginal effect on GEM-mediated cell death of Panc-1 cells. We then sought to deplete *RUNX2* using siRNA in Panc-1 cells and examined its effect on GEM sensitivity. Under our experimental conditions, *RUNX2* knockdown caused a significant enhancement of GEM sensitivity of Panc-1 cells. Notably, GEM-mediated induction of TAp63 but not of TAp73 was further stimulated in *RUNX2*-depleted Panc-1 cells, indicating that, like AsPC-1 cells, TAp63 might play a pivotal role in the regulation of GEM sensitivity of Panc-1 cells. Consistent with this notion, forced expression of TAp63α in Panc-1 cells promoted cell cycle arrest and/or cell death, and massively increased luciferase activities driven by TAp63-target gene promoters such as p21^*WAF1*^ and *NOXA*. In addition, immunoprecipitation experiments indicated that RUNX2 forms a complex with TAp63 in Panc-1 cells. Taken together, our current observations strongly suggest that depletion of *RUNX2* enhances the cytotoxic effect of GEM on *p53*-mutated Panc-1 cells through the stimulation of TAp63-dependent cell death pathway even in the presence of a large amount of pro-oncogenic mutant p53, and might provide an attractive strategy to treat pancreatic cancer patients with *p53* mutations.

## INTRODUCTION

Runt-related transcription factor 2 (RUNX2), which is one of RUNX family members, has been considered to be one of the master regulators for bone development and osteoblast differentiation. Indeed, *RUNX2*-deficient mice died just after birth and displayed a complete loss of bone formation [1, 2]. Consistent with these observations, it has been shown that RUNX2 transactivates several osteoblast differentiation-related marker genes such as *osteocalcin*, *collagen type I alpha 1* and *osteopontin* [3]. In addition to its vital role in the regulation of bone formation, a growing body of evidence strongly suggests that RUNX2 has a pro-oncogenic potential. For example, RUNX2 has been shown to be associated with the progression of prostate cancer, and tightly linked to bone metastasis of breast cancer cells [4, 5]. Kuo et al. found that RUNX2 induces acute myeloid leukemia [6]. Kayed et al. described that RUNX2 is aberrantly overexpressed in pancreatic cancer and affects the tumor microenvironment [7]. In accordance with these results, Jessica et al. showed that RUNX2 promotes a tumorigenic phenotype of breast cancer and is predictive of poor overall survival of breast cancer patients [8].

In contrast to pro-oncogenic RUNX2, a nuclear transcription factor p53 is a classical tumor supppressor. Its tumor suppressive role has been shown by two independent findings. Firstly, the extensive mutation searches demonstrated that *p53* is frequently mutated in human tumor tissues (around 50%), and over 90% of its mutations are detected within the genomic region encoding its sequence-specific DNA-binding domain, implying that these p53 mutants lack the sequence-specific tranactivation ability and thereby losing its pro-apoptotic function. The sequence-specific transactivation ability of p53 is tightly linked to its cell death-inducing function. Moreover, p53 mutants exhibit a dominant-negative behaviour against wild-type p53, and also acquire pro-oncogenic potential [9, 10]. Secondary, *p53*-deficient mice developed spontaneous tumors [11]. Collectively, both of these observations strongly support the notion that p53 is a representative tumor suppressor.

Of note, *p53* mutation has been detectable in approximately 75% of human pancreatic cancer [12], which shows the worst prognosis among human tumors (5-year survival rate is less than 5%) [13]. For chemotherapy, DNA damaging agent gemcitabine (GEM) is a current first-line of the standard treatment given to the most patients with advanced and metastatic pancreatic cancer [14–16], however, its efficacy is quite limited [17]. Since the complete surgical resection of pancreatic cancer is difficult due to its difficulty in early detection [18], chemotherapy, radiotherapy and/or immunotherapy is a remaining option. Therefore, it is urgent to clarify the molecular basis behind GEM-resistant phenotype of pancreatic cancer and also develop a novel strategy to improve clinical outcomes of patients with this deadly disease.

Meanwhile, p53 is a member of a small pro-apoptotic p53 family including p53, p73 and p63. As expected from their structures, p73/p63 acts as a nuclear transcription factor to transactivate a overlapping set of p53-target genes implicated in the induction of cell cycle arrest (*p21^WAF1^* and *14-3-3σ*), cellular senescence (*p21^WAF1^*) and/or cell death (*BAX*, *NOXA* and *PUMA*) [19, 20]. *p73/p63* encodes two major varients such as TA and ΔN isoforms, arising from alternative splicing and promoter usage, respectively. TA isoform contains an NH_2_-terminal transactivation domain and has a sequence-specific transactivation ability. In contrast to TA isoform, transcription-deficient ΔN isoform lacks an NH_2_-terminal transactivation domain. Like p53, TAp73/TAp63 becomes activated in response to DNA damage, and promotes tumor cell death [21]. It is worth noting that p53-dependent cell death following DNA damage requires TAp73 and/or TAp63, whereas TAp73 and/or TAp63 induces DNA damage-mediated cell death in the absence of p53 [22]. Unlike *p53*, *p73*/*p63* is rarely mutated in human tumors [23]. Thus, it is highly likely that TAp73 and/or TAp63 might promote DNA damage-mediated cell death of tumor cells lacking functional p53.

Intriguingly, we have recently found for the first time that siRNA-mediated silencing of *RUNX2* in *p53*-proficient human osteosarcoma U2OS cells augments their adriamycin (ADR)-sensitivity in a p53/TAp73-dependent manner [24, 25]. In addition, we have also demonstrated that GEM sensitivity of *p53*-null human pancreatic cancer AsPC-1 cells is further enhanced by *RUNX2* knockdown through the stimulation of TAp63-dependent cell death pathway [26], which was consistent with the findings that forced expression of TAp73 promotes cell cycle arrest and/or cell death in AsPC-1 cells [27]. Based on our recent results, RUNX2 markedly attenuated the transcriptional as well as pro-apoptotic activity of p53 in response to DNA damage through the complex formation with HDAC6 and p53 [24], and also significantly reduced GEM sensitivity of *p53*-deficient pancreatic cancer cells through the suppression of TAp63 expression [26]. Therefore, our recent findings indicate that RUNX2 might abrogate the proper DNA damage response in the above-mentioned *p53*-proficient and/or *p53*-null tumor cells through the inhibition of p53 family-dependent cell death pathway. The remaining question is that RUNX2 could contribute to drug-resistant phenotype of *p53*-mutated tumor cells.

In this study, we have found that *RUNX2* depletion-mediated further induction of TAp63 improves the cytotoxic effect of GEM on *p53*-mutated Panc-1 cells (R273H), and thus our present observations strongly suggest that the disruption of the balance between the intracellular endogenous amounts of pro-oncogenic mutant p53 and pro-apoptotic TAp63 might be one of the attractive strategies for the enhancement of GEM efficacy of pancreatic cancer patients.

## RESULTS

### *p53*-mutated human pancreatic cancer Panc-1 cells display gemcitabine (GEM)-resistant phenotype

**Figure 1:**
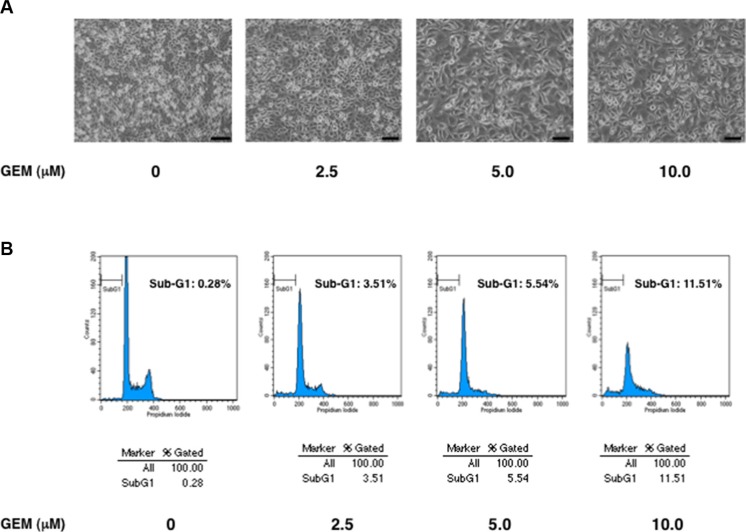
*p53*-mutated Panc-1 cells show a poor response to gemcitabine (GEM) (**A**) Phase-contrast micrographs. Panc-1 cells were treated with the indicated concentrations of GEM. Forty-eight hours after treatment, representative pictures were taken. (**B**) Flow cytometric analysis. Panc-1 cells were treated as in A. Forty-eight hours after treatment, the attached and floating cells were collected and subjected to flow cytometric analysis.

Firstly, we have assessed gemcitabine (GEM) sensitivity of *p53*-mutated human pancreatic cancer Panc-1 cells. To this end, Panc-1 cells were exposed to the indicated concentrations of GEM. Forty-eight hours after treatment, pictures of cells were taken (Figure 1A). Close inspection of them showed that GEM-treated Panc-1 cells are larger in size and number of the attached cells are reduced in response to GEM. We then collected the attached and floating cells and performed flow cytometric analysis. As shown in Figure 1B, percentage of cells with sub-G1 DNA content (dead cells) was around 10% at 10 μM of GEM. These results were also supported by trypan blue exclusion assay, which also demonstrated that GEM treatment suppresses cell proliferation rate in a dose-dependent manner (Figure S1).

Considering that approximately 20% of *p53*-proficient human pancreatic cancer SW1990 cells undergo cell death under the same experimental conditions [26], it is suggestive that Panc-1 cells are much more resistant to GEM relative to SW1990 cells. In accordance with these observations, Panc-1 cells have been considered to be one of drug-resistant pancretaic cancer cells [28]. Since Panc-1 cells express mutant p53, it is likely that GEM-resistant phenotype of Panc-1 cells might be attributed at least in part to the presence of mutant p53. In support of this notion, it has been well documented that mutant p53 causes an enhanced metastatic potential and anti-cancer drug-resistance of malignant tumor cells [29, 30].

### GEM-mediated induction of TAp73/TAp63 together with their target gene products in Panc-1 cells

**Figure 2:**
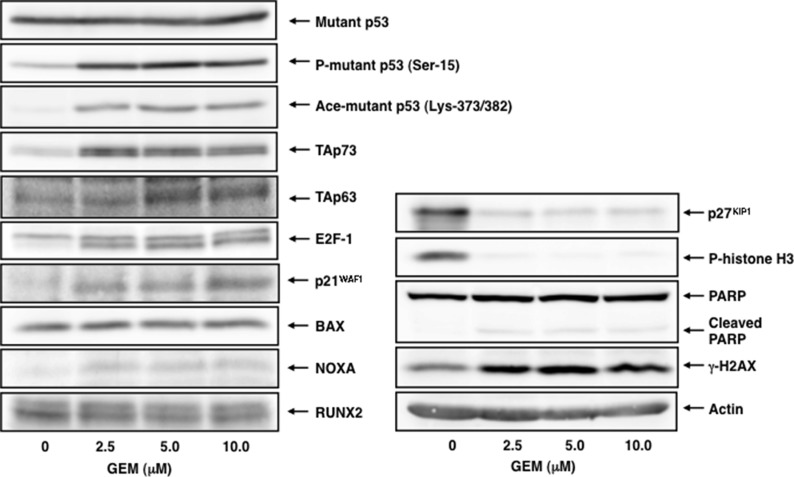
GEM-mediated induction of TAp73/TAp63 together with their target gene products Immunoblotting analysis. Panc-1 cells were treated with GEM as in Figure 1A. Forty-eight hours after treatment, whole cell lysates were prepared and processed for immunoblotting with the indicated antibodies. Actin was used as a loading control.

To understand the molecular events occurred in Panc-1 cells following GEM exposure, we have carried out immunoblotting experiments using whole cell lysates prepared from Panc-1 cells exposed to the indicated concentrations of GEM. As seen in Figure 2, mutant p53 was stably overexpressed regardless of GEM treatment, which was consistent with the results obtained from indirect immunofluorescence staining and the semi-quantitative RT-PCR experiments under normal condition (Figure S2).

Since the amounts of γH2AX was remarkably increased after GEM treatment, GEM introduced DNA damage into genomic DNA of Panc-1 cells. Meanwhile, GEM treatment markedly reduced the amounts of p27^KIP1^ and phospho-histone H3 at Ser-10, which are the reliable mitosis markers [31, 32], whereas a proteolytic cleavage of PARP, which is one of cell death markers, was barely detectable after GEM exposure, indicating that GEM treatment suppresses cell proliferation rate but not efficiently promotes cell death.

For p53 family, TAp73/TAp63 was induced in response to GEM in association with an up-regulation of their target gene products such as p21^WAF1^ and NOXA. Nakaya et al. also revealed that GEM treatment induces p21^WAF1^ expression in Panc-1 cells [33]. Consistent with these observations, E2F-1, a transcriptional activator for *TAp73* [34, 35], was up-regulated following GEM exposure. Similar results were also obtained from the semi-quantitative RT-PCR analysis (Figure S3). As mentioned above, *p53*-mutated Panc-1 cells were much more resistant to GEM as compared to *p53*-proficient SW1990 cells. Based on our present results, it is possible that a large amount of mutant p53 might prohibit the sufficient induction of TAp73/TAp63 and/or attenuate their pro-apoptotic activity following GEM exposure. The expression level of pro-oncogenic RUNX2 protein was basically constant at mRNA and protein level regardless of GEM exposure.

### Mutant *p53* knockdown has a marginal effect on GEM-mediated cell death of Panc-1 cells

**Figure 3:**
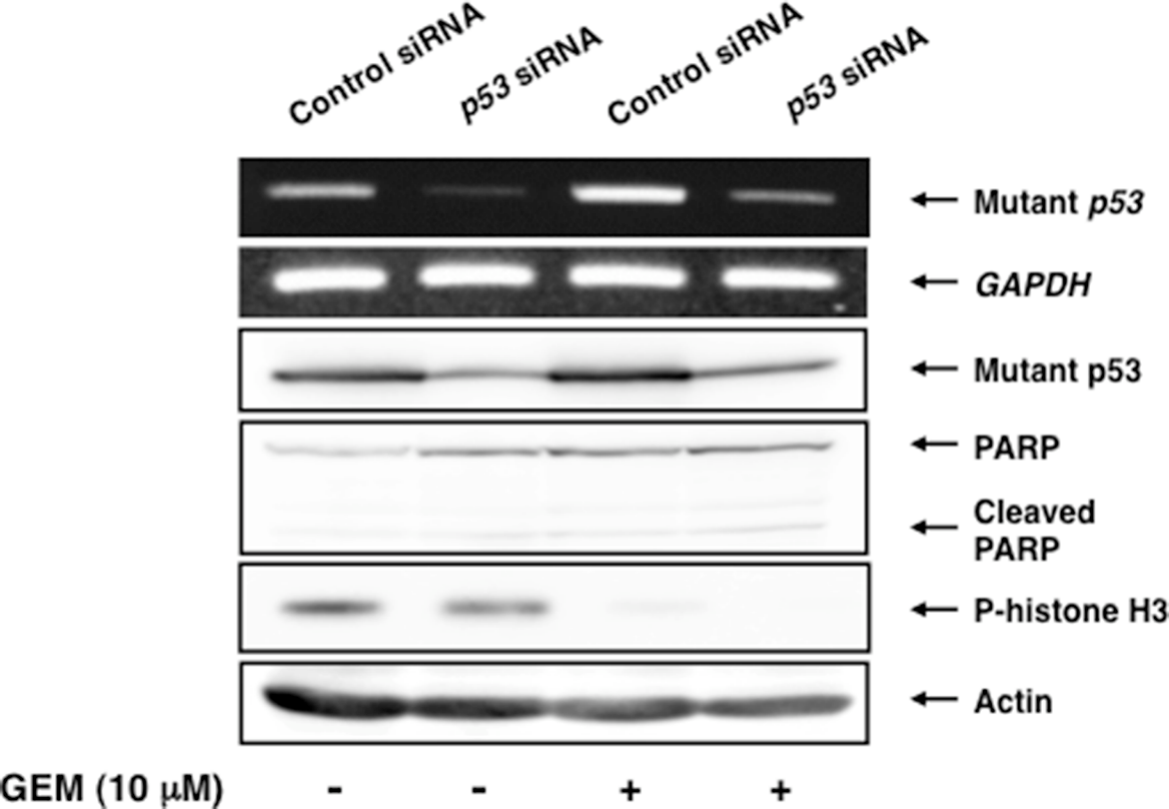
Depletion of mutant *p53* does not stimulate GEM-mediated proteolytic cleavage of PARP Panc-1 cells were transiently transfected with control siRNA or with siRNA towards *p53*. Twenty-four hours post transfection, cells were treated with 10 μM of GEM or left untreated. Forty-eight hours after treatment, total RNA and whole cell lysates were extracted and analyzed by semi-quantitative RT-PCR analysis and immunoblotting, respectively. *GAPDH* and actin were used as an internal and a loading control, respectively.

**Figure 4:**
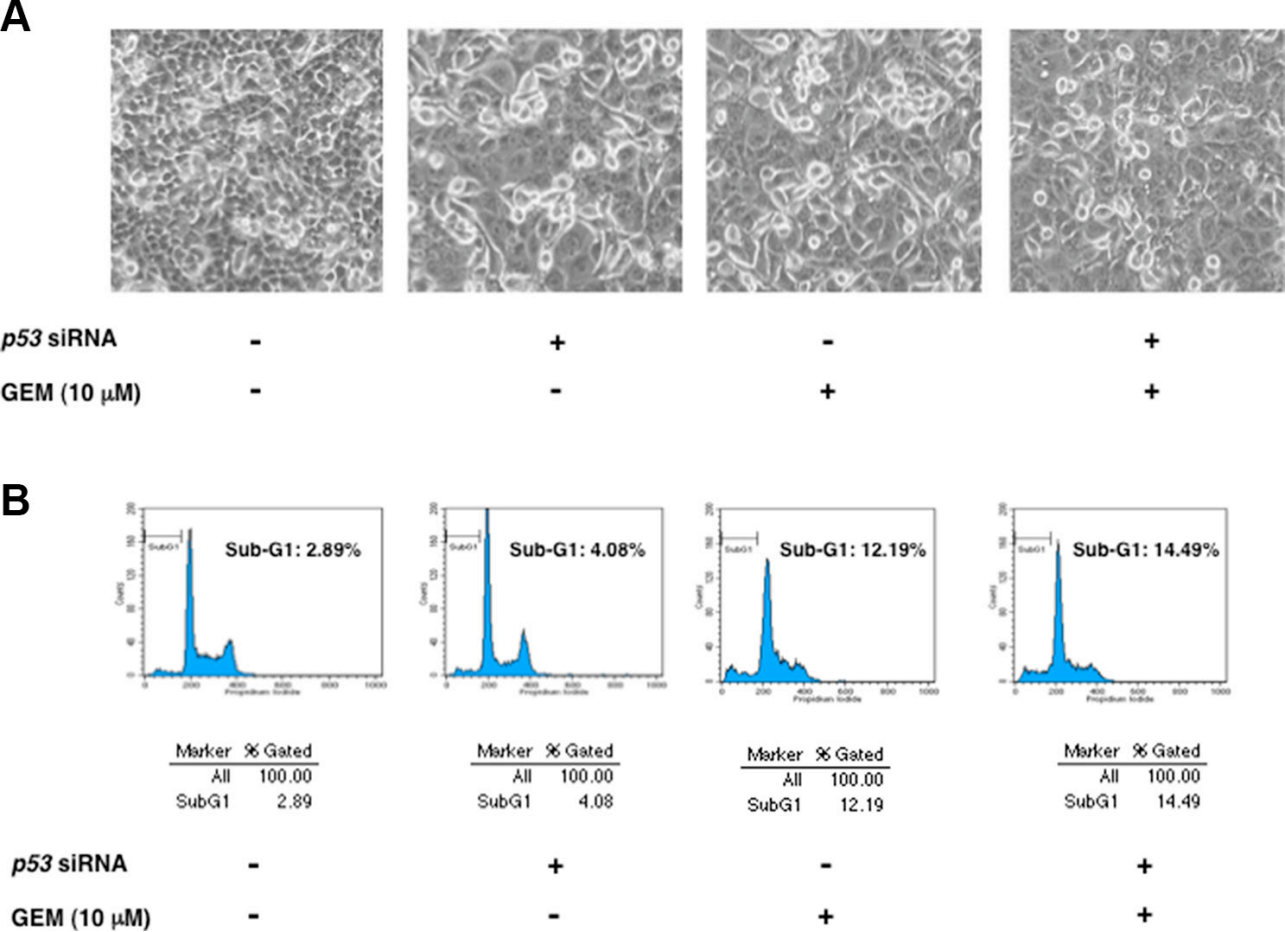
Knockdown of mutant *p53* has a marginal effect on GEM-mediated cell death (**A**) Phase-contrast micrographs. Panc-1 cells were transiently transfected with control siRNA or with siRNA against *p53*. Twenty-four hours after transfection, cells were treated with 10 μM of GEM or left untreated. Forty-eight hours after treatment, representative pictures were taken. (**B**) Flow cytometric analysis. Panc-1 cells were treated as in A. Forty-eight hours post GEM exposure, the adherent and floating cells were harvested and their DNA content was measured by flow cytometric analysis.

To verify the possibility that pro-apoptotic activity of TAp73/TAp63 could be prohibited by a large amount of mutant p53 expressed in Panc-1 cells, we sought to deplete mutant *p53* by siRNA-mediated knockdown. Since Panc-1 cells do not carry wild-type *p53* allele [36], we have employed siRNA targeting wild-type *p53* to knockdown mutant *p53* in these experiments. As shown in Figure 3, our siRNA efficiently reduced the expression of mutant p53 both at mRNA and protein levels. As clearly seen in Figure 4A, a marked morphological change (large in size) and an evident decrease in number of viable cells were observed in mutant *p53*-knockdown Panc-1 cells (Figure S4), which might be associated with cell cycle arrest [37]. Additionally, the amounts of phopho-histone H3 at Ser-10 was slightly decreased in mutant *p53*-depleted Panc-1 cells, implying that proliferation rate of Panc-1 cells slows down (Figure 3).

Unexpectedly, depletion of mutant *p53* had an undetectable effect on GEM-induced proteolytic cleavage of PARP (Figure 3). Consistent with these results, we did not observe a massive increase in number of floating cells and cells with sub-G1 DNA content in mutant *p53*-depleted Panc-1 cells exposed to GEM (Figure 4A and 4B). Similar results were also obtained from trypan blue exclusion assay (Figure S4). Collectively, it is conceivable that depletion of mutant *p53* alone is not sufficient to enhance the cytotoxic effect of GEM on Panc-1 cells.

### Knockdown of *RUNX2* augments GEM-mediated cell death of Panc-1 cells

**Figure 5:**
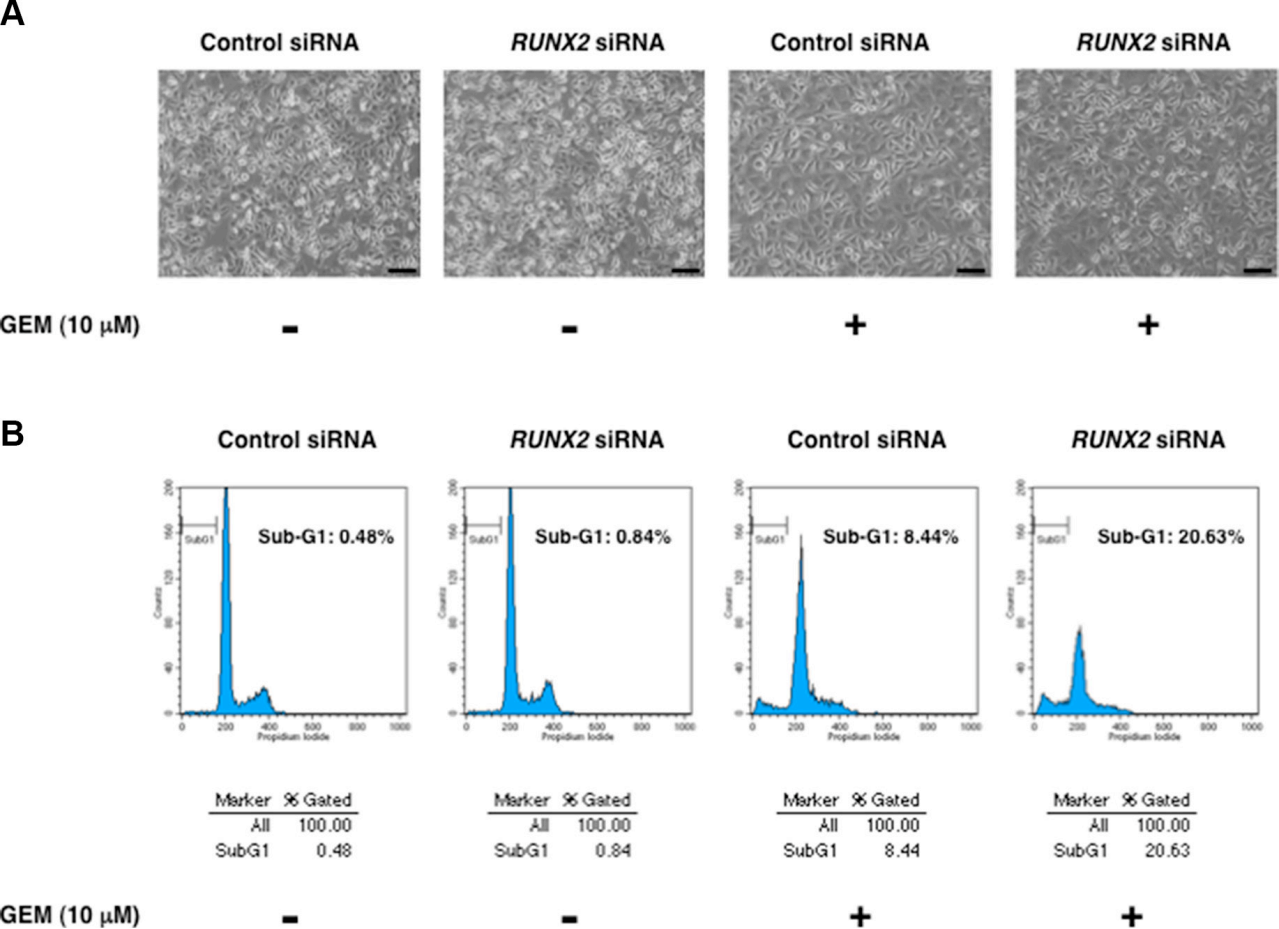
*RUNX2*-depleted Panc-1 cells efficiently undergo cell death in response to GEM (**A**) Phase-contrast micrographs. Panc-1 cells were transiently transfected with control siRNA or with siRNA against *RUNX2*. Twenty-four hours after transfection, cells were treated with 10 μM of GEM or left untreated. Forty-eight hours after treatment, representative pictures were taken. (**B**) Flow cytometric analysis. Panc-1 cells were treated as in A. Forty-eight hours post GEM exposure, the adherent and floating cells were collected and their DNA content was measured by flow cytometric analysis.

Recently, we have found for the first time that RUNX2 attenuates p53 family-dependent cell death pathway of *p53*-proficient and/or *p53*-deficient tumor cells in response to DNA damage [25, 26]. These observations prompted us to ask whether silencing of *RUNX2* could also improve the cytotoxic effect of GEM on *p53*-mutated Panc-1 cells in a TAp73/TAp63-dependent manner. For this purpose, Panc-1 cells were transfected with control siRNA or with siRNA against *RUNX2* and then treated with GEM or left untreated. As seen Figure 5A and 5B, number of the attached cells was markedly reduced and cells with sub-G1 DNA content was remarkably increased in *RUNX2*-depleted cells exposed to GEM as compared to that of control cells treated with GEM. Similar results were also obtained from trypan blue exclusion and DNA fragmentation assays (Figure S5 and S6), indicating that knockdown of *RUNX2* improves the cytotoxic effect of GEM. While, indirect immunostaining experiments revealed that silencing of *RUNX2* has a marginal effect on number of Ki-67-positive cells (Figure S7).

**Figure 6:**
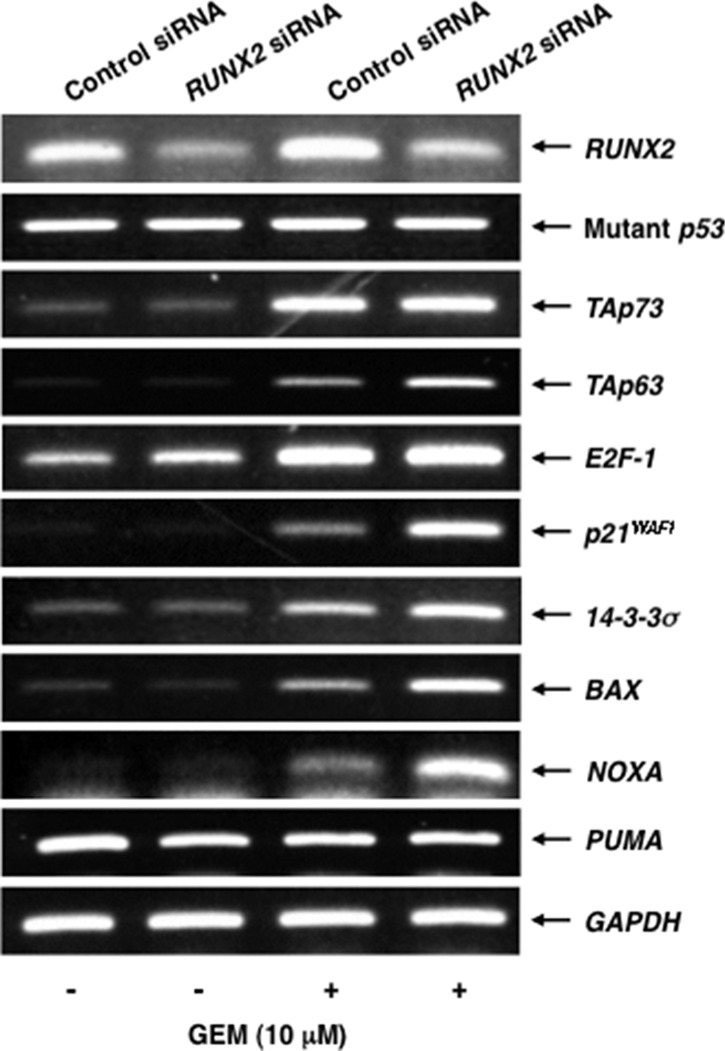
Silencing of *RUNX2* in Panc-1 cells further stimulates GEM-mediated induction of *TAp63* and its target gene expression Panc-1 cells were treated as in Figure 5A. Forty-eight hours after GEM exposure, total RNA was prepared and analyzed by semi-quantitative RT-PCR. *GAPDH* was used as an internal control.

Next, we have examined the expression patterns of p53 family-related genes in *RUNX2*-depleted Panc-1 cells in response to GEM. Under our experimental conditions, *RUNX2* knockdown was successful at mRNA level (Figure 6). The expression levels of mutant *p53* and p53 family-target gene *PUMA* remained unchanged regardless of GEM exposure together with or without *RUNX2* knockdown. As shown also in Figure S3, *TAp73* and its transcriptional activator gene *E2F-1* were induced in response to GEM, while *RUNX2* knockdown had an undetectable effect on GEM-mediated induction of *TAp73* as well as *E2F-1*. Notably, silencing of *RUNX2* further augmented GEM-mediated stimulation of *TAp63* and p53 family-target genes such as *p21^WAF1^*, *14-3-3σ*, *BAX* and *NOXA*.

**Figure 7:**
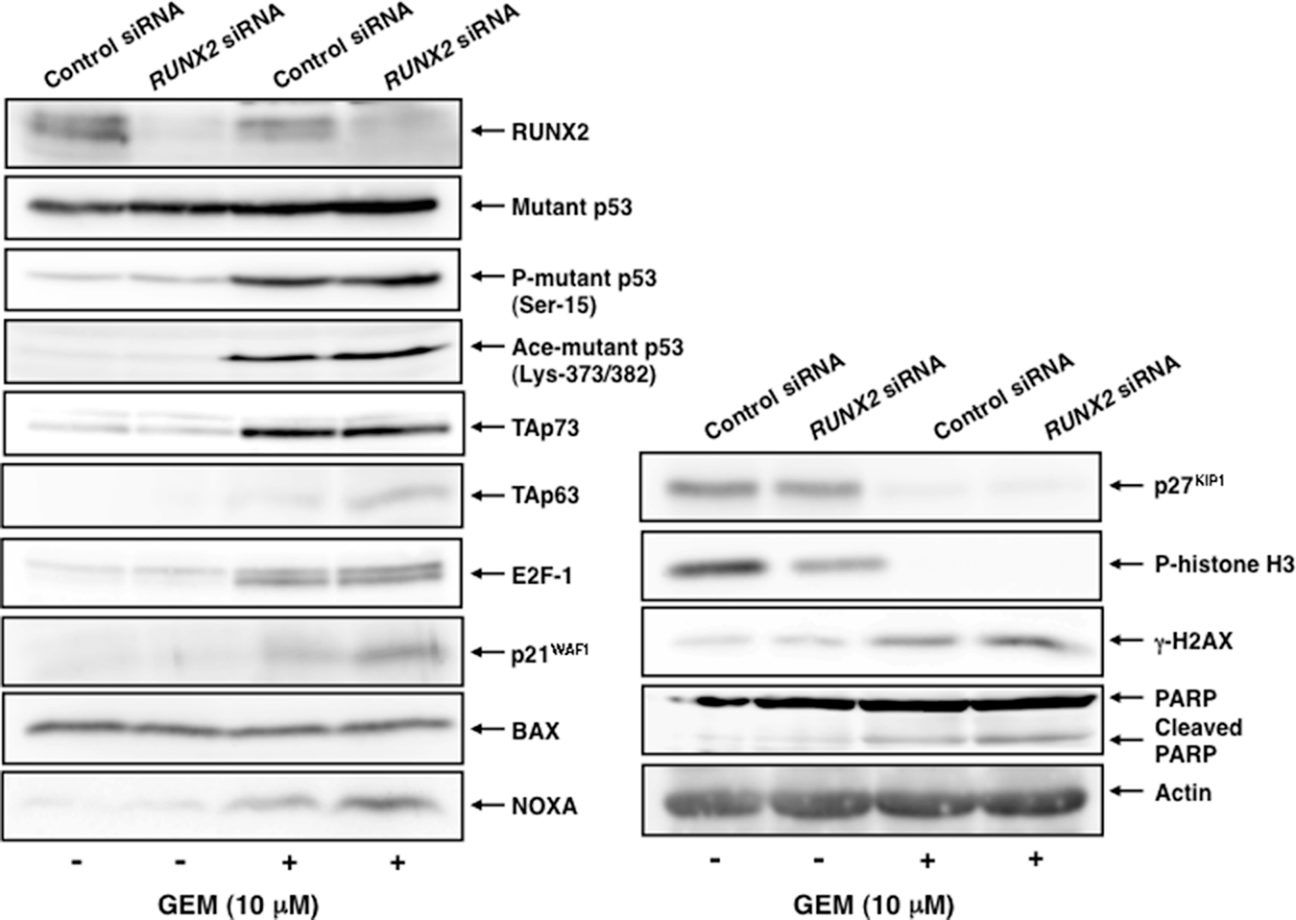
GEM-mediated induction of TAp63 and its target gene products is further augmented in *RUNX2*-depleted Panc-1 cells Panc-1 cells were transfected and treated with GEM as in Figure 5A. Forty-eight hours after GEM exposure, whole cell lysates were prepared and subjected to immunoblotting with the indicated antibodies. Actin was used as a loading control.

To confirm the results obtained from the semi-quantitative RT-PCR analysis, we performed immunoblotting analysis. As shown in Figure 7, mutant p53 and p53 family-target gene product BAX remained constant regardless of GEM exposure with or without *RUNX2* knockdown. Similarly, pro-apoptotic PUMA remained unchanged in *RUNX2*-depleted Panc-1 cells exposed to GEM relative to untreated Panc-1 cells (data not shown). GEM-mediated up-regulation of phosphorylation of mutant p53 at Ser-15, acetylation at Lys-373/382, TAp73 and E2F-1 was detectable, whereas *RUNX2* silencing-dependent further accumulation of them was not observed. As expected, GEM-mediated induction of TAp63 and p53 family-target gene products including cell cycle-related p21^WAF1^ and pro-apoptotic NOXA was further augmented in *RUNX2*-depleted Panc-1 cells. Thus, these observations suggest that depletion of *RUNX2* enhances the cytotoxic effect of GEM on Panc-1 cells through the up-regulation of TAp63-dependent cell death pathway.

### Forced expression of TAp63 promotes cell cycle arrest and/or cell death of Panc-1 cells

**Figure 8:**
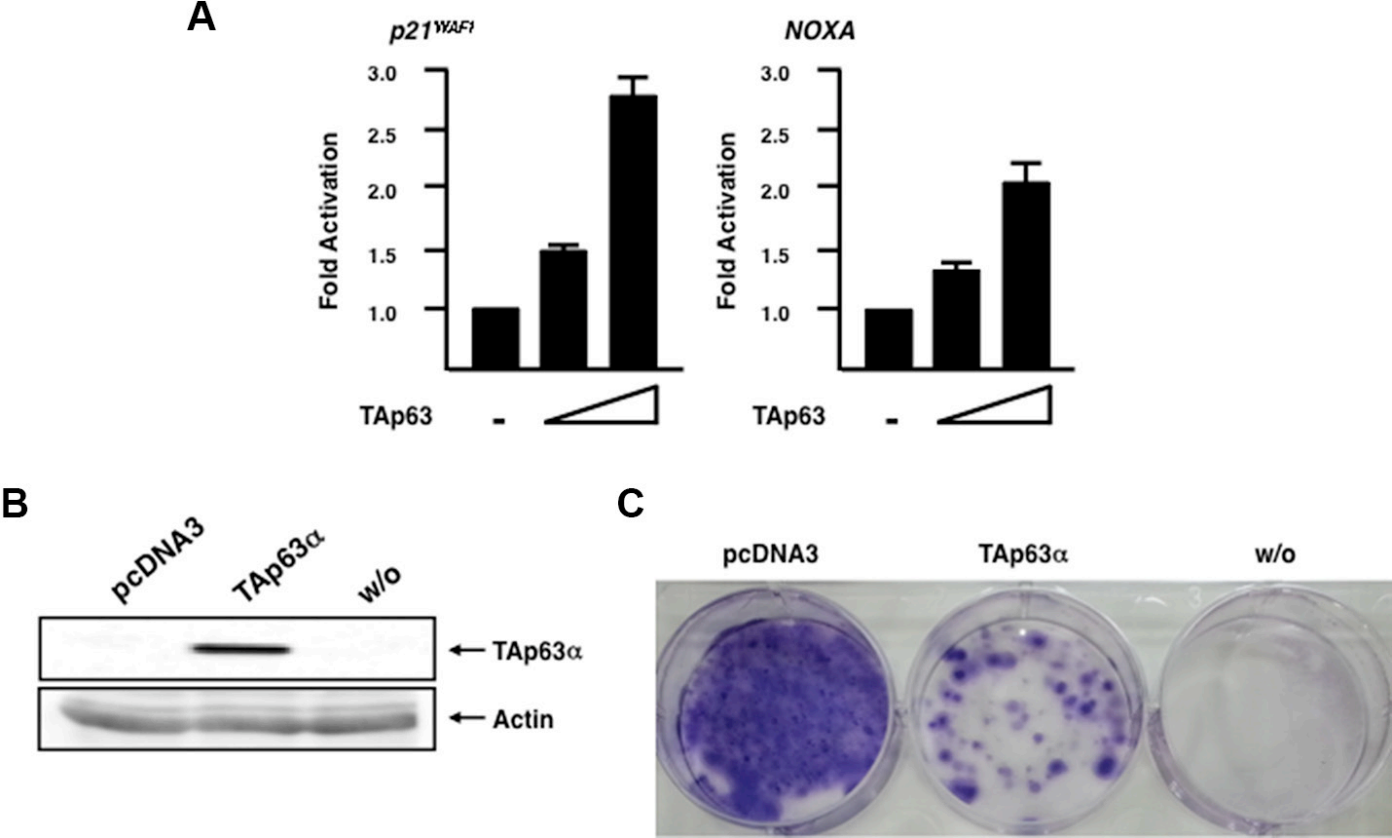
Forced expression of TAp63α in Panc-1 cells enhances p53 family-target promoter activities and promotes cell cycle arrest and/or cell death (**A)** Luciferase reporter assay. Panc-1 cells were transiently transfected with the constant amount of luciferase reporter plasmid carrying human *p21^WAF1^* or *NOXA* promoter (100 ng) and *Renilla* luciferase plasmid (10 ng) along with or without the increasing amounts of the expression plasmid for TAp63α(100 or 200 ng). Total amount of plasmid DNA was kept constant with pcDNA3 (510 ng). Forty-eight hours after transfection, cell lysates were prepared and their luciferase activities were measured. (**B)** Exogenous expression of TAp63α. Panc-1 cells were transfected with the empty plasmid or with the expression plasmid encoding TAp63α. Forty-eight hours after transfection, whole cell lysates were prepared and analyzed by immunoblotting with anti-p63 antibody. Actin was used as a loading control. (**C)** Panc-1 cells were transfected as in B. Forty-eight hours after transfection, cells were transferred to the fresh medium containing 800 μg/mL of G418. Two weeks after the selection, G418-resistant colonies were fixed with methanol and stained with Giemsa's solution.

To ask whether forced expression of TAp63 alone could enhance p53 family-target promoter activities in Panc-1 cells, we carried out luciferase reporter assay. To this end, Panc-1 cells were transfected with the luciferase reporter plasmid carrying human p53 family-responsible *p21^WAF1^* or *NOXA* promoter and *Renilla* luciferase plasmid together with or without the increasing amounts of TAp63α (the longest TAp63 isoform) [20] expression plasmid. Forty-eight hours after transfection, cell lysates were prepared and their luciferase activities were measured. As clearly seen in Figure 8A, luciferase activity driven by *p21^WAF1^* or *NOXA* promoter was markedly elevated in TAp63α-overexpressing Panc-1 cells in a dose-dependent manner, indicating that forced expression of TAp63 transactivates its target gene promoters even in the presence of mutant p53.

We then asked whether forced expression of TAp63 could also promote cell cycle arrest and/or cell death in Panc-1 cells. For this purpose, Panc-1 cells were transfected with the empty plasmid (pcDNA3) or with the expression plasmid for TAp63α. Forty-eight hours after transfection, cells were transferred into fresh medium containing G418. Two weeks after selection, G418-resistant colonies were fixed and stained with Giemsa's solution. As shown in Figure 8B, forced expression of TAp63α was successful. As expected, number of G418-resistant colonies was significantly reduced in TAp63α-overexpressing cells as compared to that in pcDNA3-transfected cells (Figure 8C), indicating that TAp63 induces cell cycle arrest and/or cell death in Panc-1 cells.

### Silencing of *p63* suppresses GEM-dependent DNA fragmentation

**Figure 9:**
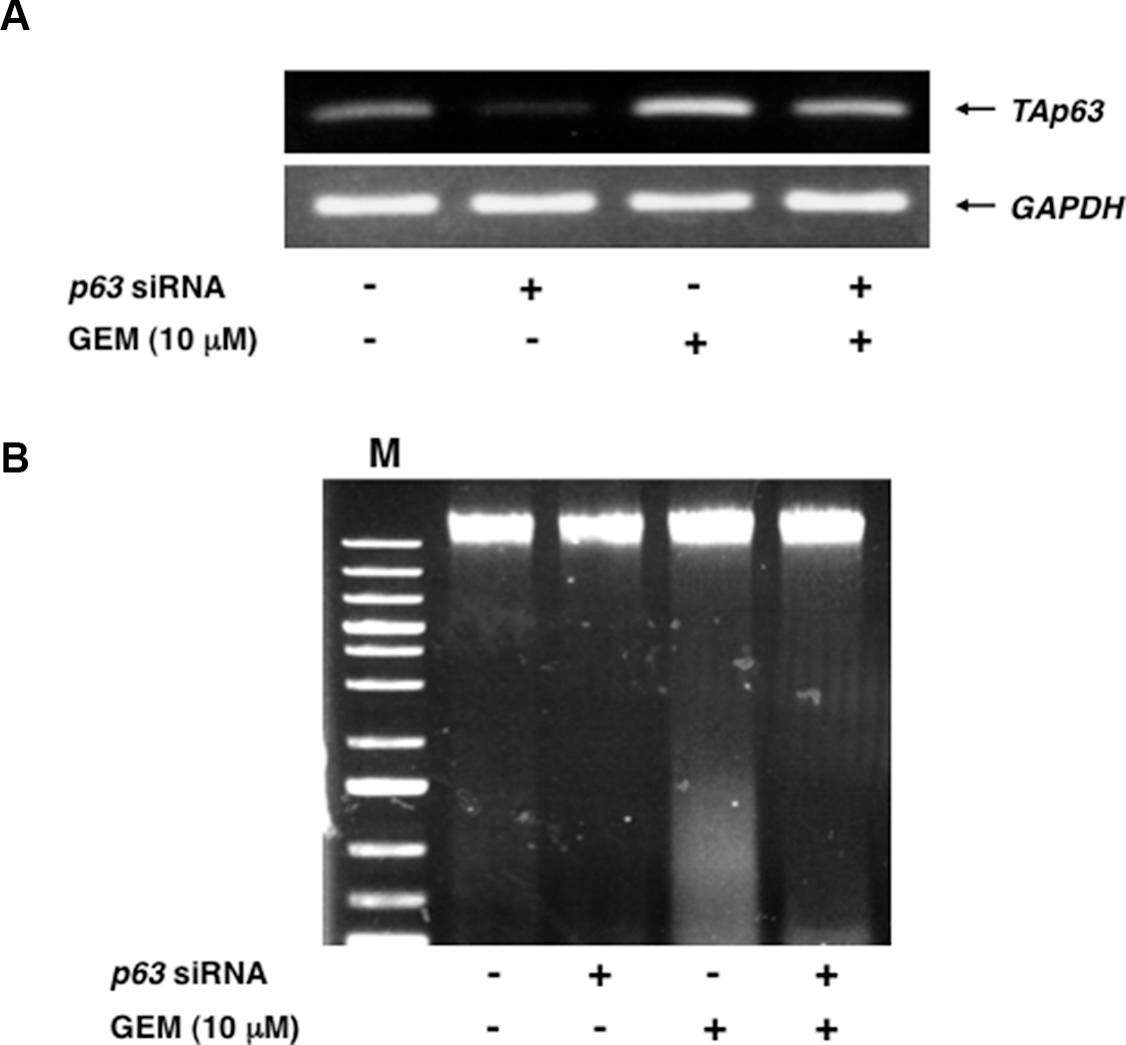
Silencing of *p63* attenuates GEM-mediated DNA fragmentation (**A**) Knockdown of *p63*. Panc-1 cells were transfected with control siRNA or with siRNA against *p63*. Twenty-four hours after transfection, cells were exposed to 10 μM of GEM or left untreated. Forty-eight hours after treatment, total RNA was isolated and analyzed by semi-quantitative RT-PCR. *GAPDH* was used as an internal control. (**B**) DNA fragmentation assay. Panc-1 cells were treated as in A. Forty-eight hours after treatment, floating and attached cells were harvested, their genomic DNA was prepared and subjected to agarose electrophoresis. M indicates the size marker.

To verify whether RUNX2/TAp63 regulatroy axis could play a vital role in GEM-induced cell death of Panc-1 cells, we have conducted siRNA-mediated knockdown of *p63*. Panc-1 cells were transfected with control siRNA or with siRNA targeting *p63* and then treated with or without GEM. As seen in Figure 9A, knockdown of *TAp63* was successful. Under the same experimental conditions, genomic DNA was prepared from the indicated cells and analyzed by 0.7% agarose gel electrophoresis. As expected, GEM-dependent DNA fragmentation was significantly attenuated in *p63*-depleted cells as compared to non-depleted cells (Figure 9B).

Together, our present observations strongly suggest that depletion of *RUNX2* improves the cytotoxic effect of GEM on *p53*-mutated Panc-1 cells through the stimulation of TAp63-dependent cell death pathway.

## DISCUSSION

In the present study, we have found for the first time that siRNA-mediated silencing of *RUNX2* enhances the cytotoxic effect of GEM on *p53*-mutated pancreatic cancer Panc-1 cells through the augmentation of TAp63-dependent cell death pathway. Indeed, exogenous expression of TAp63α in Panc-1 cells increased the luciferase activities driven by p53 family-target promoters and also promoted cell cycle arrest and/or cell death as examined by luciferase reporter analysis and colony formation assay, respectively. As described [28], Panc-1 cells are the typical drug-resistant pancreatic cancer cells. Thus, it is highly likely that intracellular balance of the endogenous expression levels between pro-apoptotic TAp63 and pro-oncogenic mutant p53 might be a vital determinant of pancreatic cancer cell fate in response to GEM.

It has been well established that RUNX2 acts as one of the master transcriptional regulators of bone development through the sequence-specific transactivation of *osteocalcin*, *collagen type I alpha 1* and *osteopontin* [3]. Accumulating evidence, however, strongly suggests that RUNX2 also has a pro-oncogenic potential [4–8]. In accordance with this notion, several lines of evidence demonstrated that, in addition to osteoblast differentiation-related genes, RUNX2 transactivates invasion and metastasis-related genes such as *MMP*s (*metalloproteinases*) and *VEGF* (*vascular endothelial growth factor*) [38–40]. Indeed, Tandon et al. described that RUNX2 promotes metastatic spread of mammary tumor cells and depletion of *RUNX2* abrogates the late-stage tumor cell growth [41]. Of note, Lim et al. found that RUNX2 transactivates pro-oncogenic *survivin* and thus contributes to the growth of prostate tumor [42]. Recently, Trotter et al. demonstrated that RUNX2 is tightly involved in the promotion of multiple myeloma growth, survival and progression through the up-regulation of Akt/beta-catenin/survivin pro-oncogenic signaling pathway [43]. Under our experimental conditions, we have observed that survivin expression is siginificantly elevated in Panc-1 cells exposed to GEM, indicating that survivin contributes at least in part to GEM-resistant phenotype of Panc-1 cells (data not shown). As described [44], *survivin* expression was negatively regulated by wild-type p53. Since Panc-1 cells lack wild-type p53, it is possible that *survivin* might be escaped from wild-type p53-mediated transcriptional repression machinery. Intriguingly, GEM-mediated stimulation of survivin was attenuated in *RUNX2*-depleted Panc-1 cells (data not shown). In view of the observed expression pattern of survivin during GEM exposure, further studies should be required to clarify the molecular mechanisms of the possible involvement of RUNX2/survivin pro-oncogenic pathway in the genesis and/or maintenance of GEM-resistant phenotype of pancreatic cancer cells. Based on our present observations, knockdown of *RUNX2* in Panc-1 cells further stimulated GEM-mediated induction of cell cycle-related p21^WAF1^ and pro-apoptotic NOXA, which was tightly associated with up-regulation of TAp63. These results prompted us to ask whether both of p53 family-target gene products (p21^WAF1^ and NOXA) could be required for GEM-mediated cell death. Our additional experiments revealed that silencing of *NOXA* causes a poor response to GEM in Panc-1 cells, whereas GEM-dependent cell death is obviously enhanced in *p21^WAF1^*-depleted Panc-1 cells (Figure S8). These observations suggest that TAp63-mediated induction of NOXA but not of p21^WAF1^ is essential for GEM-dependent cell death of Panc-1 cells.

As mentioned above, silencing of *RUNX2* in Panc-1 cells caused a significant induction of TAp63 at mRNA and protein level. Consistent with our present observations, we have recently demonstrated that *TAp63* is up-regulated in *RUNX2*-depleted pancreatic cancer AsPC-1 cells, and also found the putative RUNX2-binding consensus sites within the 5′-upstream region of *TAp63* gene (26). Although further studies should be required to adequately address this issue, it is possible that RUNX2-mediated down-regulation of *TAp63* might be one of the molecular mechanisms behind GEM-resistant phenotype of Panc-1 cells. As seen in Figure 8, forced expression of TAp63α in Panc-1 cells stimulated the luciferase activity driven by p53 family-target promoters such as *p21^WAF1^* and *NOXA* and also induced cell cycle arrest and/or cell death even in the presence of mutant p53. These observations imply that a large amount of TAp63 overcomes the negative effect of mutant p53 as well as RUNX2 against TAp63. Intriguingly, our immunoprecipitation experiments revealed that RUNX2 forms a complex with TAp63 in Panc-1 cells (Figure S9). In addition to the possible RUNX2-mediated transcriptional repression of *TAp63*, it is conceivable that RUNX2 prohibits the transcriptional as well as pro-apoptotic function of TAp63 through the complex formation with TAp63.

Since silencing of *RUNX2* improved the cytotoxic effect of GEM on Panc-1 cells, it is of potential interest to investigate the proteolytic degradation system of RUNX2. It is possible that GEM-resistant phenotype of Panc-1 cells might be at least in part due to the basically constant expression level of RUNX2 in response to GEM. Jonason et al. described that RUNX2 is tightly regulated at transcription level and also through post-translational mechanisms involving the ubiquitin-proteasome pathway [45]. In this connection, it has been shown that E3 ubiquitin ligases such as Smurf1, WWP1 and CHIP promote ubiquitin/proteasome-mediated degradation of RUNX2 [46–48]. In addition to them, Kumar et al. found that there is an inverse relationship between the expression patterns of RUNX2 and E3 ubiquitin ligase Fbw7α, and Fbw7α promotes ubiquitination-dependent proteasomal degradation of RUNX2 [49]. Notably, several lines of evidence showed that Fbw7 acts as a tumor suppressor by targeting numerous pro-oncogenic proteins for ubiquitination and degradation such as c-Myc, c-Jun, Mcl-1 and cyclin E [50, 51]. Moreover, Ji et al. described that loss of function of Fbw7 is closely implicated in the development of pancreatic cancer [52]. Therefore, it is of great interest to further investigate whether there could exist a functional link among TAp63, RUNX2 and Fbw7 in response to GEM.

Another finding of our present study is that, unexpectedly, knockdown of mutant *p53* alone is not sufficient to further enhance GEM-mediated cell death of Panc-1 cells. From our present results, depletion of mutant *p53* markedly suppressed cell proliferation rate as examined by trypan blue exclusion assay. Similarly, Zhu et al. found that siRNA-mediated silencing of mutant *p53* in human bladder cancer T24 and 5637 cells leads to a massive growth inhibition [28]. In addition, cisplatin (CDDP) sensitivity was evidently enhanced in mutant *p53*-depleted bladder cancer cells [26]. It might be attributed to CDDP-mediated activation of wild-type p53 encoded by the remaining allele of these bladder cancer cells, however, Panc-1 cells lack wild-type *p53* allele [35]. Ge et al. revealed that re-activation of wild-type p53 in *p53*-mutated prostate cancer DU145 cells induces cell cycle arrest [53, 54].

Zhu et al. also demonstrated that, in addition to the suppression of cell proliferation rate caused by silencing of mutant *p53*, cell death as well as G2-phase cell cycle arrest simultaneously takes place in mutant *p53*-depleted bladder cancer cells [28]. When compared to control siRNA-transfected Panc-1 cells, mutant *p53*-depleted Panc-1 cells were larger in size and exhibited a lower proliferation rate accompanied by a slight decrease in the amounts of phospho-histone H3 at Ser-10. Since phospho-histone H3 at Ser-10 is one of the established molecular markers of mitosis, mitotic arrested cells and/or cells with prolonged mitosis accumulate phospho-histone H3 at Ser-10 [31]. Considering that the lower proliferation rate and the slight decrease in the amounts of phospho-histone H3 at Ser-10 in mutant *p53*-knockdown Panc-1 cells, it is suggestive that knockdown of mutant *p53* alone might induce cell cycle arrest before the initiation and/or after the termination of mitosis. Alternatively, it has been shown that phospho-histone H3 at Ser-10 is essential for neoplastic cell transformation and tumor development independent on cell cycle profile [55, 56]. Further studies should be required to adequately address the possible role of phospho-histone H3 at Ser-10 in the sensitivity to GEM.

In conclusion, our current findings strongly suggest that depletion of *RUNX2* improves GEM sensitivity of *p53*-mutated pancreatic cancer Panc-1 cells in a TAp63-dependent manner, and thus our present study might provide a novel strategy to enhance the cytotoxic efficacy of GEM on aggressive and metastatic pancreatic cancer patients with *p53* mutation.

## MATERIALS AND METHODS

### Cell culture

Human pancreatic cancer Panc-1 cells were maintained in Dulbecco's modified Eagle's medium (DMEM; WAKO, Osaka, Japan) supplemented with 10% heat-inactivated fetal bovine serum (FBS; Invitrogen, Carlsbad, CA, USA), 100 U/mL penicillin and 50 μg/mL streptmycin in incubators with humidified atmospheres of 5% CO_2_ and 95% air at 37°C.

### WST assay

Cells were transferred into 96-well plates at a density of 1 × 10^3^/well and incubated overnight. After the incubation, cells were exposed to the indicated concentrations of gemcitabine (GEM) (WAKO, Osaka, Japan). Forty-eight hours after treatment, the relative number of viable cells was assessed using Cell Counting Kit-8 reagent (Dojindo, Kumamoto, Japan) according to the manufacturer's instructions. Experiments were performed in triplicate.

### Trypan blue exclusion assay

The effect of GEM on cell proliferation was analyzed by the standard trypan blue exclusion assay. In brief, cells were seeded at a density of 5 × 10^5^/6-well plates and cultured overnight. After the incubation, cells were treated with the indicated concentrations of GEM. Forty-eight hours after treatment, the attached and floating cells were collected, mixed with the equal volume of 0.4% trypan blue solution (Bio-Rad Laboratories, Hercules, CA, USA) and subjected to automated assessment using a TC-20 automated cell counter (Bio-Rad Laboratories).

### FACS analysis

For flow cytometric analysis, cells were exposed to the indicated concentrations of GEM. Forty-eight hours after treatment, the attached and floating cells were harvested and fixed in 70% ethanol at −20°C overnight. After fixation, cells were incubated with 1 mg/mL of RNase A at 37°C for 30 min, and stained with 50 mg/mL of propidium iodide (PI). There DNA contents were analyzed by a FACScan flow cytometer equipped with Cell Quest software (BD Biosciences, San Jose, CA, USA) according to the manufacturer's protocols.

### Colony formation assay

Cells were tranfected with the expression plasmid for TAp63α or with the empty plasmid pcDNA3 (Invitrogen, Carlsbad, CA, USA) using Lipofectamine 2000 transfection reagent (Invitrogen) according to the manufacturer's suggestions. Forty-eight hours after transfection, cells were transferred to the fresh medium containing G418 (at a final concentration of 800 mg/mL). Two weeks after the selection, G418-resistant colonies were fixed with methanol and stained with Giemsa's solution (Merck, Darmstadt, Germany).

### Semi-quantitative RT-PCR

Total RNA was extracted from GEM-treated or untreated cells using RNeasy mini kit (Qiagen, Hilden, Germany) according to the manufacturer's instructions. One microgram of total RNA was reverse transcribed by SuperScript VILO cDNA synthesis system (Invitrogen) according to the manufacturer's protocols. The resultant cDNA was used for PCR-based amplification. Oligonucleotide primer sets used in this study were as follows: *p53*, 5′-CTGCCCTCAACAAGATGTTTTG-3′ (forward) and 5′-CTATCTGAGCAGCGCTCATGG-3′ (reverse); *TAp63*, 5′-GACCTGAGTGACCCCATGTG-3′ (forward) and 5′-CGGGTGATGGAGAGAGAGCA-3′ (reverse); *TAp73*, 5′- TCTGGAACCAGACAGCACCT-3′ (forward) and 5′- GTGCTGGACTGCTGGAAAGT*-3*′ (reverse); *RUNX2*, 5′-TCTGGCCTTCCACTCTCAGT-3′ (forward) and 5′-GACTGGCGGGGTGTAAGTAA-3′ (reverse); *p21^WAF1^*, 5′-ATGAAATTCACCCCCTTTCC-3′ (forward) and 5′-CCCTAGGCTGTGCTCACTTC-3′ (reverse); *14-3-3σ*, 5′-GAGCGAAACCTGCTCTCAGT-3′ (forward) and 5′-CTCCTTGATGAGGTGGCTGT-3′ (reverse); *NOXA*, 5′-CTGGAAGTCGAGTGTGCTACT-3′ (forward) and 5′-TCAGGTTCCTGAGCAGAAGAG-3′ (reverse); *PUMA*, 5′-GCCCAGACTGTGAATCCTGT-3′ (forward) and 5′-TCCTCCCTCTTCCGAGATTT-3′ (reverse); *BAX*, 5′-AGAGGATGATTGCCGCCGT-3′ (forward) and 5′-CAACCACCCTGGTCTTGGAT-3′ (reverse); *GAPDH*, 5′-ACCTGACCTGCCGTCTAGAA-3′ (forward) and 5′-TCCACCACCCTGTTGCTGTA-3′ (reverse). *GAPDH* expression was examined as an internal control. PCR products were separated on 1.5% agarose gels and visualized by ethidium bromide staining.

### Immunoblotting

Cells were washed twice in ice-cold phosphate-buffered saline (PBS) and lysed in lysis buffer containing 25 mM Tris-HCl, pH 8.0, 137 mM NaCl, 2.7 mM KCl, and 1% Triton X-100 supplemented with a commercial protease inhibitor mixture (Sigma, St. Louis, MO, USA). Whole cell lysates (50 μg of protein) were analyzed by immunoblotting as described [24] using the following antibodies: anti-p53 (DO-1; Santa Cruz Biotechnology, Santa Cruz, CA, USA), anti-phospho-p53 at Ser-15 (Cell Signaling Technologies, Beverly, MA, USA), anti-acetyl-p53 at Lys-373/382 (Millipore, Billerica, MA, USA), anti-TAp63 (N2C1, GeneTex, Irvine, CA, USA), anti-p21^WAF1^ (H164; Santa Cruz Biotechnology), anti-BAX (Cell Signaling Technologies), anti-NOXA (Cell Signaling Technologies), anti-E2F-1 (Cell Signaling Technologies), anti-RUNX2 (Cell Signaling Technologies), anti-p27^KIP1^ (Cell Signaling Technologies), anti-phospho-histone H3 at Ser-10 (Cell Signaling Technologies), anti-PARP (Cell Signaling Technologies), anti-γH2AX (2F3; BioLegend, San Diego, CA, USA) or anti-actin antibody (20–33; Sigma). The immuno-reactive proteins were visualized using enhanced chemiluminescence (GE Healthcare Life Sciences, Buckinghamshire, United Kingdom) according to the manufacturer's instructions.

### Immunopresipitation

Panc-1 cells were transfected with the expression plasmid for TAp63α. Forty-eight hours after transfection, cell lysates were immunoprecipitated with normal mouse serum (NMS) or with anti-RUNX2 antibody (8G5, Medical and Biological Laboratories, Nagoya, Japan). The immuno-precipitates were analyzed by immunoblotting with anti-TAp63 antibody.

### Luciferase reporter assay

Panc-1 cells were seeded at a density of 5 × 10^4^/12-well plate and cultured overnight. Cells were then transfected with the constant amount of luciferase reporter plasmid carrying human *p21^WAF1^* or *NOXA* promoter, *Renilla* luciferase plasmid together with or without the increasing amounts of the expression plasmid for TAp63α. Total amount of plasmid DNA was kept constant with the empty plasmid pcDNA3. Forty-eight hours after transfection, cell lysates were prepared and their luciferase activities were measured using dual luciferase reporter assay system (Promega, Madison, WI, USA).

### Indirect immunofluorescence staining

The indicated cells were washed twice in ice-cold PBS, fixed in 3.7% formaldehyde for 30 min at room temperature, permeabilized with 0.2% Triton X-100 for 5 min at room temperature, and blocked with 3% bovine serum albumin (BSA) in PBS for 1 h at room temperature. After blocking, cells were washed three times in PBS and probed with anti-p53 antibody for 1 h at room temperature followed by incubation with rhodamin-conjugated goat anti-mouse IgG (Invitrogen) for 1 h at room temperature. Cells were then washed three times in PBS and cell nuclei were stained with 4′,6-diamidino-2-phenylindole (DAPI). Images were captured using a confocal laser scanning microscope (Leica, Wetzlar, Germany).

### DNA fragmentation assay

High molecular weight genomic DNA was prepared according to the standard procedure. After RNase A treatment, genomic DNA (100 ng) was analyzed by 0.7% agarose gel electrophoresis, and visualyzed by ethidium bromide staining.

### Statistical analysis

All of the data are presented as the mean ± SD. Student's two-tailed *t*-test was employed for our study, and a *p* value less than 0.05 was considered statistically significant.

## SUPPLEMENTARY MATERIALS FIGURES



## Abbreviations

BSA: bovine serum albumin
DAPI: 4′6-diamidino-2-phenylindole
FACS: fluorescence activated cell sorting
FBS: fetal bovine serum
GAPDH: glyceraldehyde 3-phosphate dehydrogenase
GEM: gemcitabine
MMP: metalloproteinase
PBS: phosphate-buffered saline
PARP: poly ADP ribose polymerase
PI: propidium iodide
RUNX2: runt-related transcription factor 2
siRNA: small interfering RNA.
