# Supplementary material for: Depletion of pro-oncogenic RUNX2 enhances gemcitabine (GEM) sensitivity of p53-mutated pancreatic cancer Panc-1 cells through the induction of pro-apoptotic TAp63
Source: Oncotarget. 2016 Oct 4;7(44):71937–50. doi: 10.18632/oncotarget.12433 (PMC5342134; doi:10.18632/oncotarget.12433)
Supplement: Supplementary file 1 [file oncotarget-07-71937-s001.pdf]

Depletion of pro-oncogenic *RUNX2* enhances gemcitabine (GEM) sensitivity of *p53*-mutated pancreatic cancer Panc-1 cells through the induction of pro-apoptotic TAp63

Supplementary Materials

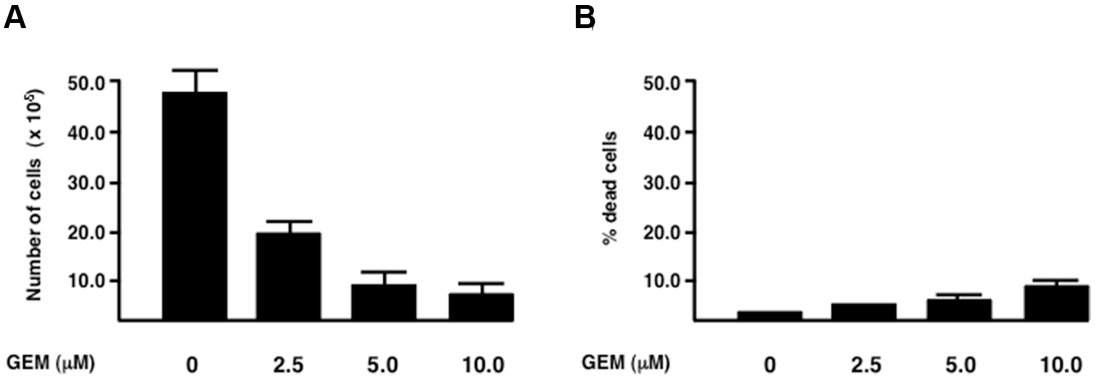

Supplementary Figure S1: Panc-1 cell proliferation is strongly prohibited by GEM, whereas GEM-mediated cell death is barely detectable. (**A**) and (**B**) Panc-1 cells were treated with GEM as in Figure 1A. Forty-eight hours after treatment, cells were stained with trypan blue and then processed for the standard trypan blue exclusion assay. Number of unstained cells (viable cells) and % stained cells (dead cells) were shown in A and B, respectively.

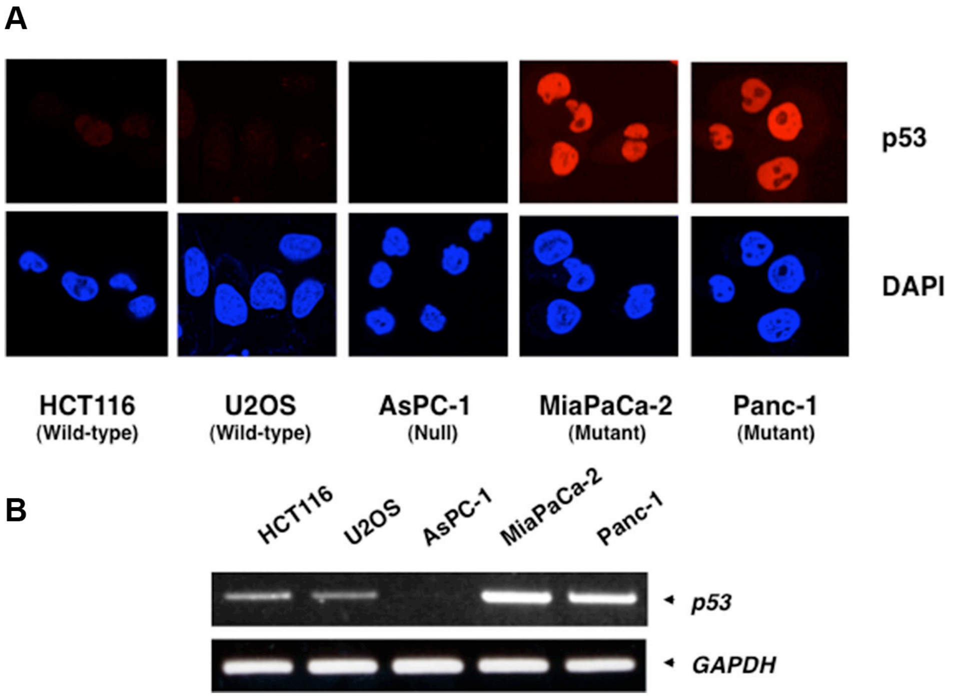

Supplementary Figure S2: Stable overexpression of mutant p53 in MiaPaCa-2 and Panc-1 cells. (**A**) Indirect immunofluorescence staining. Human colon cancer HCT116, osteosarcoma U2OS, pancreatic cancer AsPC-1, MiaPaCa-2 and Panc-1 cells were fixed in formaldehyde and stained with anti-p53 antibody (*red*). Cell nuclei were stained with DAPI (*blue*). (**B**) Total RNA was extracted from the indicated cancerous cells and analyzed for the expression level of wild-type *p53* (HCT116 and U2OS) and mutant *p53* (MiaPaCa-2 and Panc-1). *GAPDH* was used as an internal control.

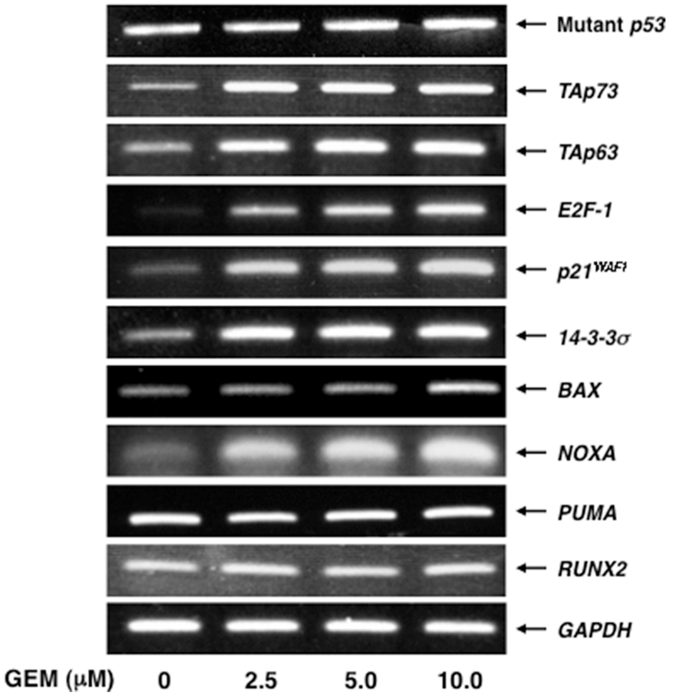

Supplementary Figure S3: *TAp73/TAp63* together with their target genes are induced in response to GEM. Semi-quantitative RT-PCR analysis. Panc-1 cells were treated with GEM as in Figure 1A. Forty-eight hours after treatment, total RNA was prepared and analyzed by semi-quantitative RT-PCR. *GAPDH* expression levels were examined as an internal control.

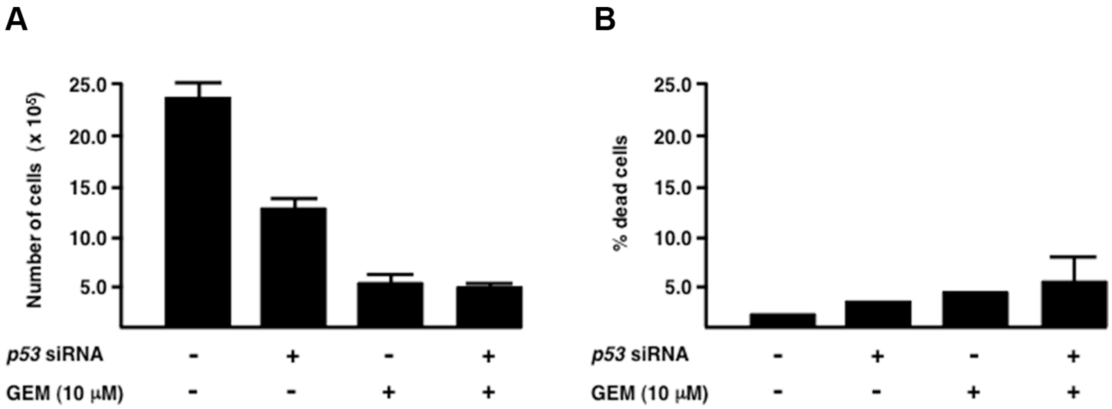

Supplementary Figure S4: Knockdown of mutant *p53* in Panc-1 cells induces growth retardation but does not siginificantly promote cell death. (**A**) and (**B**) Panc-1 cells were transfected and treated with GEM as in Figure 3. Forty-eight hours after treatment, cells were stained with trypan blue and then subjected to the standard trypan blue exclusion assay. Number of unstained cells (viable cells) and % stained cells (dead cells) were shown in A and B, respectively.

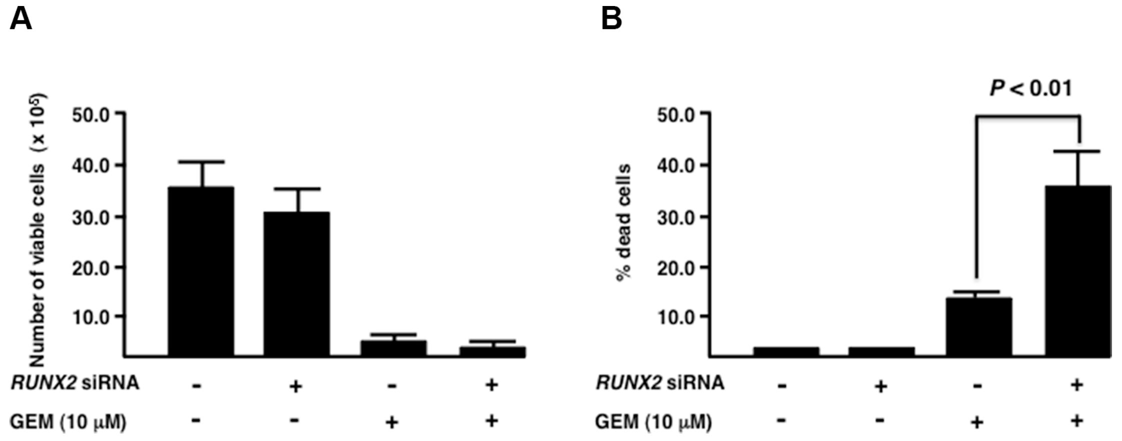

Supplementary Figure S5: Depletion of *RUNX2* in Panc-1 cells further promotes GEM-mediated cell death. (**A**) and (**B**) Panc-1 cells were transfected and treated with GEM as in Figure 5A. Forty-eight hours after treatment, cells were stained with trypan blue and then subjected to the standard trypan blue exclusion assay. Number of unstained cells (viable cells) and % stained cells (dead cells) were shown in A and B, respectively.

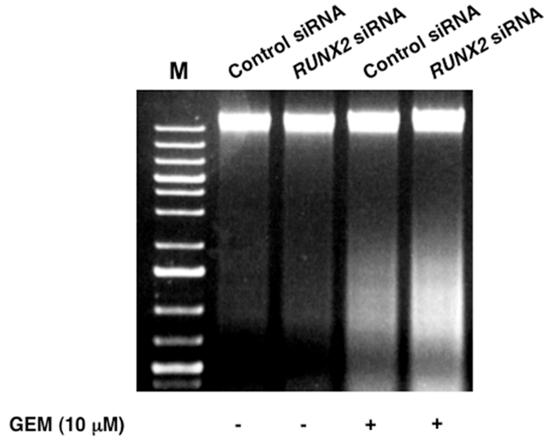

Supplementary Figure S6: *RUNX2* depletion enhances GEM-mediated DNA fragmentation. Panc-1 cells were transfected with control siRNA or with siRNA towards *RUNX2* and treated with or without 10 μM of GEM. Forty-eight hours after treatment, genomic DNA was prepared and subjected to 0.7% agarose gel electrophoresis. M indicates the size marker.

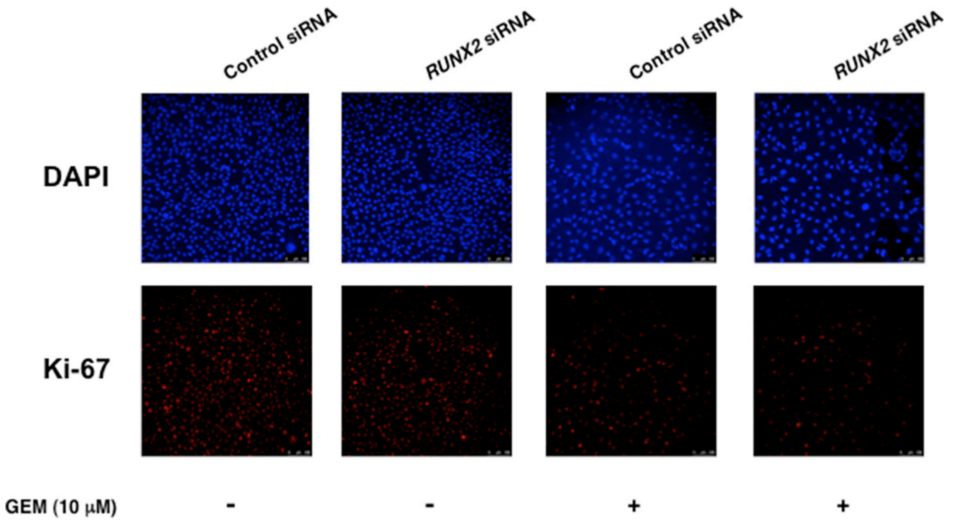

Supplementary Figure S7: Knockdown of *RUNX2* has a negligible effect on GEM-mediated growth suppression. Panc-1 cells were treated as in Supplementary Figure S6. Forty-eight hours after treatment, cells were fixed in formaldehyde and then incubated with anti-Ki-67 antibody (*red*). Cell nuclei were stained with DAPI (*blue*).

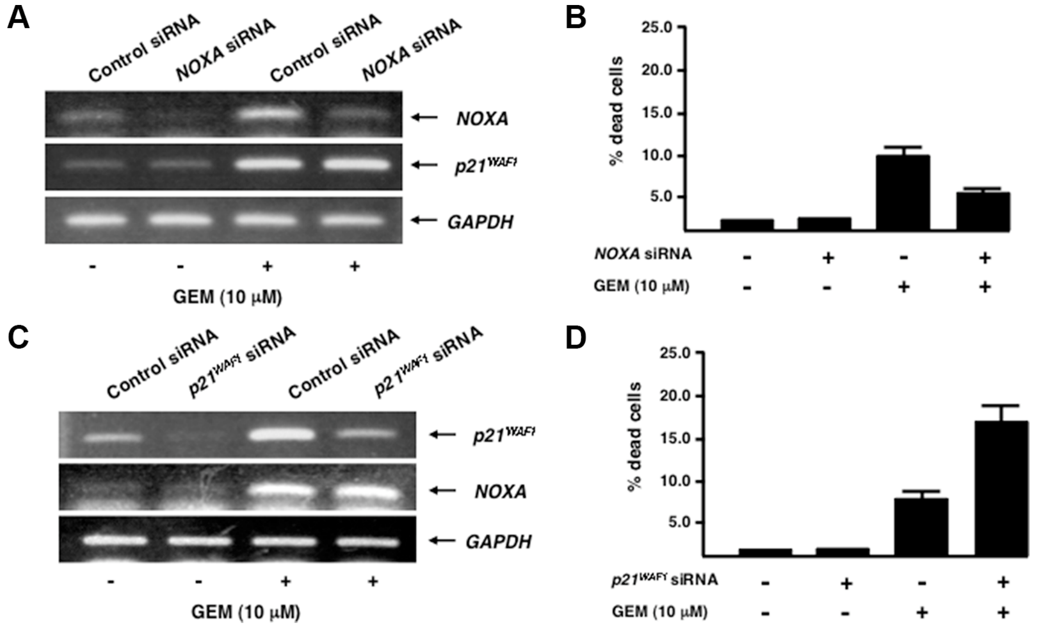

Supplementary Figure S8: NOXA but not p21^{WAF1} is required for GEM-induced cell death. Panc-1 cells were transfected with control siRNA, siRNA targeting *NOXA* (**A** and **B**) or with siRNA against *p21^{WAF1}* (**C** and **D**). Twenty-four hours after transfection, cells were treated with 10 μM of GEM or left untreated. Forty-eight hours after treatment, total RNA was prepared and analyzed by RT-PCR (A and C). Under the same experimental conditions, cells were exposed to 10 μM of GEM. Forty-eight hours after treatment, cells were sujected to trypan blue exclusion assays (B and D).

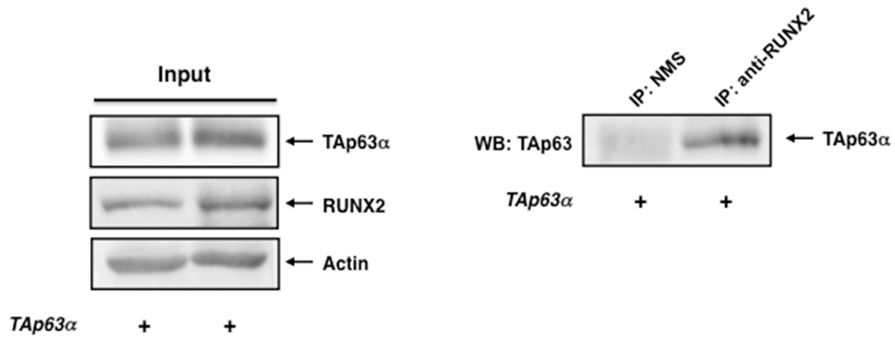

Supplementary Figure S9: Complex formation between RUNX2 and TAp63. Panc-1 cells were transfected with the expression plasmid for TAp63α. Forty-eight hours after transfection, whole cell lysates were prepared and subjected to immnoprecipitation with normal mouse serum (NMS) or with monoclonal anti-RUNX2 antibody. The immunoprecipitaes were then analyzed by immunoblotting with anti-TAp63 antibody (right panel). Left panel shows 1/20 of input.
